# Predicting Mortality in Severe Burns: A Comparison of Four Mortality Prediction Scores and the Role of Organizational Changes in the Croatian Burn Center

**DOI:** 10.3390/ebj5040036

**Published:** 2024-11-15

**Authors:** Agata Skunca, Ana Mesic, Dorotea Zagorac, Mirela Dobric, Vedran Lokosek, Morana Banic, Aleksandra Munjiza, Aisa Muratovic

**Affiliations:** 1 Department of Anesthesiology, Intensive Care and Pain Medicine, Sestre Milosrdnice University Hospital Center, 10000 Zagreb, Croatia; ana.mesic1@gmail.com (A.M.); dorotea.zagorac@kbcsm.hr (D.Z.); mirela.dobric@kbcsm.hr (M.D.); vedran.lokosek@kbcsm.hr (V.L.); morana.banic@kbcsm.hr (M.B.); aisa.muratovic@kbcsm.hr (A.M.); 2 Department of Traumatology, Sestre Milosrdnice University Hospital Center, 10000 Zagreb, Croatia; aleksandra.munjiza@kbcsm.hr

**Keywords:** burn score, mortality, severe burns, burn care

## Abstract

Background: The primary aim of this study was to evaluate the performance of four burn prognostic scores—Abbreviated Burn Severity Index (ABSI), Ryan, Belgium Outcome Burn Injury (BOBI), and revised Baux score (rBaux) in a Croatian burn center. A secondary aim was to compare patient outcomes before and after the organizational and protocol changes. Methods: A retrospective study and comparison of four prediction scores was conducted over a nine-year period in burn patients with ≥20% total body surface area (TBSA) burned. Additionally, outcomes before and after organizational changes were compared. Results: A total of 149 patients were included, with the mean patient age of 54.62 ± 19.38 years, the mean of TBSA of 42.98 ± 19.90, and an overall mortality rate of 48.99%. The area under the ROC curve (AUROC) was 0.79 for the rBaux and ABSI score, 0.77 for the BOBI score, and 0.76 for the Ryan score. The duration of mechanical ventilation and length of stay (LOS) in burn intensive care units (BICU) decreased after the organizational changes, though survival rates remained similar. Conclusions: Prognostic scores are good predictors of mortality but with moderate predictive accuracy. Continuity of care in intensive care could be important for better outcomes.

## 1. Introduction

Mortality is the most important outcome in burn injury research and clinical practice [1]. Over the past decades, several mortality prediction scores have been developed. The Baux score, introduced in 1961, was one of the earliest models. It estimates the probability of death by adding the patient’s age to the percentage of TBSA burned [2]. In 1982, the Abbreviated Burn Severity Index (ABSI) score was developed. This model incorporates five variables: patient sex, age, presence of inhalation injury, presence of full-thickness burns, and percentage of TBSA burned, with each assigned a specific numeric value [3]. The Ryan score was developed following a retrospective analysis of 1665 burn patients from 1990 to 1994. This score identifies three risk factors for mortality: age over 60 years, TBSA burns over 40%, and the presence of inhalation injury [4]. The Belgium Outcome Burn Injury (BOBI) score was introduced in 2009, using data from 1999 to 2003 (5246 patients) to develop a mortality prediction model and data from 2004 (981 patients) for validation. This score is built on the foundation of the Ryan score but further stratifies patients based on age and TBSA [5]. In response to advancements, the original Baux score was revised in 2010, and inhalational injury was added to the equation, reflecting its impact on mortality [6].

Accurate mortality prediction models are helpful for clinical practice in burn care. These models help with therapeutic strategies and discussions with the family about prognosis, and they play a role in deciding palliative care. Advancements in intensive care treatment and burn management may have reduced the predictive power of scoring systems. Mortality rates in burn patients have decreased, potentially limiting the utility of older prediction models.

Our primary aim is to assess the accuracy of four prognostic scores (rBaux, ABSI, BOBI, and Ryan) in our population of severely burned patients, a group with high mortality rates that would benefit most from precise prognostication. Our secondary aim is to compare patient demographics, injury characteristics, and outcomes across two time periods: before and after the implementation of a new protocol and organizational changes.

## 2. Materials and Methods

This retrospective observational study was conducted on severely burned patients admitted to the burn intensive care unit (BICU) at the Department of Traumatology of Sestre Milosrdnice University Hospital Center in Zagreb, Croatia, from 1 January 2016 to 1 October 2024. Data were retrieved from the hospital’s database, following approval from the Hospital Ethics Committee. The Burn Department is a specialized surgical unit staffed by a multidisciplinary team, including 2 surgeons, a surgical resident, 22 nurses, and a physiotherapist. Within the Burn Department is BICU, overseen by a leading consultant anesthesiologist skilled in burn care, who manages treatment and is present daily. Outside of regular hours, the Burn Unit (including BICU) is supported by a consultant anesthesiologist providing continuous 24 h coverage. Additionally, as part of the Burn Department, a tissue bank is maintained with a laboratory dedicated to producing cultured epithelial autografts and allografts. This specialized burn center primarily serves burn patients from Croatia, with some cases referred from neighboring Bosnia and Herzegovina. The unit comprises 5 intensive care beds and 6 additional ward beds designated for burn patients. The average number of patients admitted to the BICU per year is 29, and the department performs around 128 surgical procedures annually.

The study included adult patients (18 years and older) with a TBSA burned of 20% or greater admitted to the BICU during the study period. The exclusion criteria were as follows: superficial burns, pregnant patients, and patients whose medical treatment in the BICU was incomplete during the study period.

The following data were collected for each patient: age, sex, TBSA burned, presence of inhalation injury and full-thickness burns, duration of mechanical ventilation, timing of tracheotomy, length of stay (LOS) in BICU, LOS in hospital, and outcome.

Based on the data collected, the following severity scores were calculated: rBaux, ABSI, BOBI, and Ryan scores. Additionally, we stratified data based on two different periods and compared scores and outcomes. The first period (from 2016 to 2022) was compared to the second (from 2023 to 2024) when new organizational structure and protocols were introduced.

Before implementing the new protocol, the attending anesthesiologist rotated frequently—sometimes weekly or even daily—unlike other team members who are permanently assigned to the unit. In 2023, a significant organizational change was implemented, extending the rotation of anesthesiologist intensivists dedicated to the BICU to a duration of three to six months. Throughout this period, all decisions related to intensive treatment, during and outside regular working hours, were made collaboratively with the attending anesthesiologist. The attending anesthesiologist’s responsibilities include ensuring a consistent treatment plan and strict adherence to clinical protocols during regular and after-hours shifts, thereby maintaining high standards of patient safety and care. The assignment of a dedicated anesthesiologist has improved adherence to protocols within our BICU. While these protocols reflect practices aligned with modern ICU standards, achieving consistent compliance has been challenging due to frequent fluctuations in attending anesthesiologists, particularly during after-hours shifts. This inconsistency was the main motivation for organizational changes.

Protocols that are now more consistently adhered to include a structured approach to diagnosing inhalation injuries, requiring mandatory bronchoscopy confirmation and grading of inhalation injury for all mechanically ventilated patients. Standardized dosages and durations for inhalational therapy were also established: 5000 international units of heparin six times daily for 7–10 days, along with n-acetylcysteine and a bronchodilator. Before these updates, the diagnosis and treatment of inhalation injuries did not always include injury grading, and treatment regimens varied. Additionally, because bronchoscopy was performed by different anesthesiologists, some variability in reporting inhalation injuries existed. The use of dexmedetomidine for sedation became standard practice, replacing midazolam whenever feasible, with mandatory daily sedation breaks, and an emphasis on earlier discontinuation of sedation. Previously, the most frequently used sedative was midazolam. Vitamin D supplementation and propranolol therapy were introduced as standard practice in BICU. Infection management in patients with major burns remains challenging, with the increasing incidence of infections caused by multidrug-resistant and pandrug-resistant microorganisms. Previously, lack of consistency regarding antimicrobial therapies was more frequent, and consultations concerning microbiological samples and therapy were conducted remotely via telephone with a microbiologist. After the change, bedside clinical rounds have been implemented, enabling direct consultation with both a microbiologist and an infectious disease specialist. Also, all decisions regarding antibiotic treatment, including both duration and choice of antimicrobials, were guided by the lead anesthesiologist. The goal was to shorten the time of antimicrobial therapy whenever possible. The periodic revision of microbiological data with sensitivity profiles is undertaken every 6 months, with appropriate adjustments of empirical antibiotic therapy. Efforts to maintain protocol adherence continue to be a focus for improving patient outcomes.

Statistical analysis was conducted using SAS software (version 9.4, Cary, NC, USA). Descriptive statistics were used to summarize patient characteristics. Continuous variables were expressed as means with standard deviations (SD), and as medians with interquartile ranges (IQR). Categorical variables were expressed as frequencies and percentages. The difference between the two groups was compared using Wilcoxon Rank-Sum test or Student’s *t*-test for numeric variables, and Chi-squared test for categorical variables. The predictive accuracy of each score was evaluated using the Area Under the Receiver Operating Characteristic Curve (AUROC) with a 95% confidence interval. Sensitivity, specificity, positive predictive value (PPV), and negative predictive value (NPV) were calculated to evaluate the diagnostic performance of each prognostic score on a probability level of 0.5.

## 3. Results

### 3.1. Demographic Data

A total of 149 patients were included in the study over a 9-year period. The average age of the patients was 54.62 ± 19.38 years. Of these, 114 patients (76.51%) were male, and 35 patients (23.49%) were female, with a male-to-female ratio of 3.26:1. The mean TBSA burned was 42.98 ± 19.90%. Although most patients experienced full-thickness burns (n = 132, 88.59%), the exact percentage of full-thickness was not available in medical records. Inhalational injuries were present in 70 patients (46.98%), and mechanical ventilation was required for 116 patients (77.85%) during their hospital stay. Among the ventilated patients, 45 (38.79%) underwent a tracheotomy. The average LOS in the BICU was 35.83 ± 35.53 days, and the overall hospital stay was 55.36 ± 48.80 days. A total of 76 patients (51.01%) survived, while 73 patients (48.99%) died during hospitalization. Data are shown in Table 1.

### 3.2. Comparison of Mortality Prediction Scores

**Figure 1:**
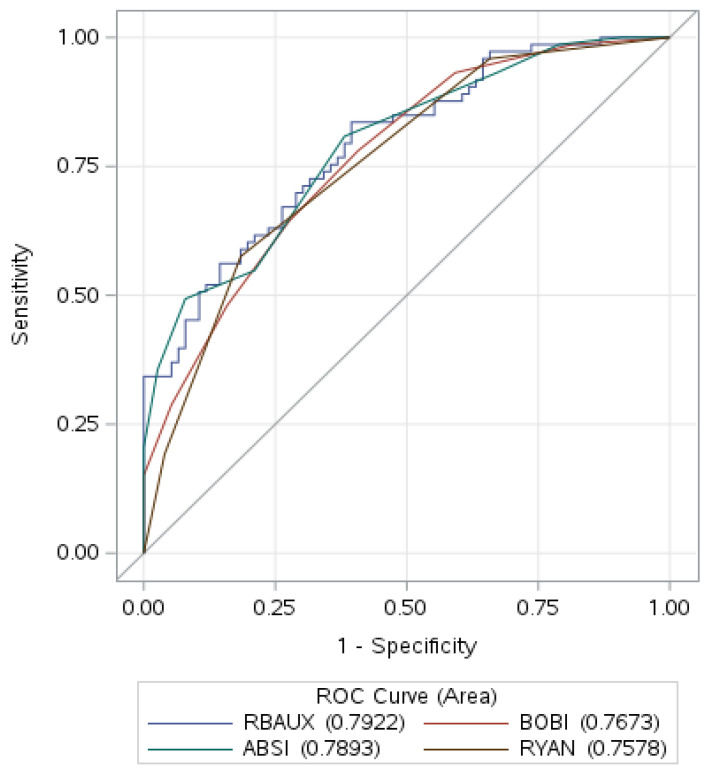
AUROC for all four scores.

The mean rBaux score for all patients was 105.69 ± 28.93, the ABSI score was 9.26 ± 2.38, the BOBI score was 4.32 ± 2.15, and the Ryan score was 1.30 ± 0.91. For patients who survived, the mean rBaux score was 91.17 ± 22.13, the ABSI score was 8.07 ± 1.75, the BOBI score was 3.38 ± 1.83, and the Ryan score was 0.88 ± 0.80. For patients who died, the mean rBaux score was 120.81 ± 27.50, the ABSI score was 10.51 ± 2.32, the BOBI score was 5.29 ± 2.02, and the Ryan score was 1.73 ± 0.82. The AUROC was 0.79 (95% CI: 0.72–0.86) for the revised Baux score, 0.79 (95% CI: 0.72–0.86) for the ABSI score, 0.77 (95% CI: 0.69–0.84) for the BOBI score, and 0.76 (95% CI: 0.69–0.83) for the Ryan score. Detailed data are shown in Table 2, and AUROC is shown in Figure 1.

### 3.3. Comparison of Two Time Periods

**Figure 2:**
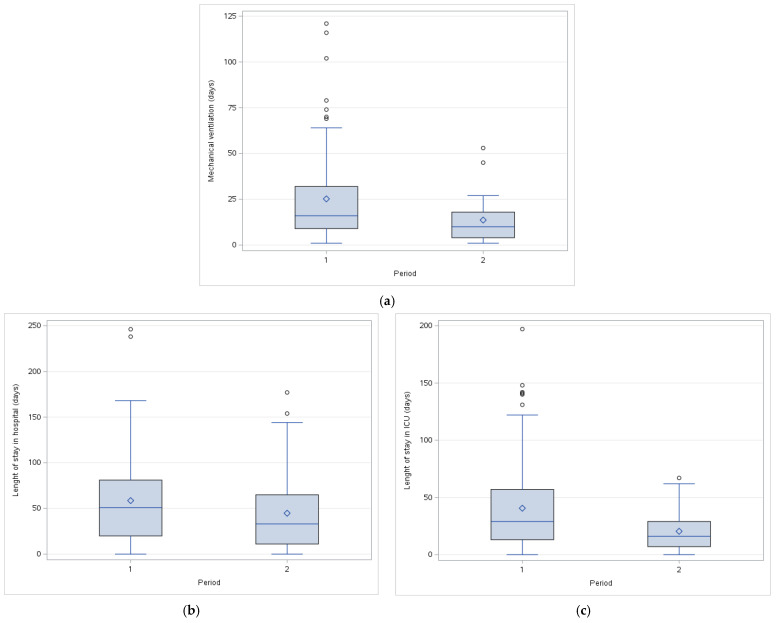
Outcomes in two different time periods; first from 2016 to 2022, and second from 2023 to 2024: (**a**) Mechanical ventilation; (**b**) Length of stay in hospital; (**c**) Length of stay in burn intensive care unit.

When comparing data from two different time periods, before and after organizational changes, the following results were observed. In the period before organizational change, the mean age of patients was 54.70 ± 20.16 years. Of these, 84 patients (73.68%) were male, and 30 patients (26.32%) were female. The mean TBSA burned was 41.99 ± 18.63%, and 100 patients (87.72%) had some degree of full-thickness burns. Inhalational injuries were present in 50 patients (43.86%). The total LOS in the BICU was 40.56 ± 38.23 days, and the hospital LOS was 58.61 ± 49.25 days. Of these patients, 58 (50.88%) survived. After the organizational change, the mean age of patients was 54.34 ± 16.82 years. Of these, 30 patients (85.71%) were male, and 5 patients (14.29%) were female. The mean TBSA burned was 46.19 ± 23.59%, and 32 patients (91.43%) had some degree of full-thickness burns. Inhalational injuries were present in 20 patients (57.14%). The total LOS in the BICU was 20.40 ± 17.82 days, and the hospital LOS was 44.77 ± 46.41 days. Of these patients, 18 (51.43%) survived. Results are shown in Table 3 and Figure 2.

## 4. Discussion

All four prognostic scores are statistically significant predictors of mortality, but the AUROC values (ranging from 0.76 to 0.79) were slightly lower than in similar studies [7,8,9,10]. This indicates that while the scores demonstrate moderate predictive accuracy for mortality, they may not be as accurate as in previous studies. The sensitivity and specificity of all four scores suggest that they are moderately effective in distinguishing between those who survived and died. The rBaux score demonstrated the highest sensitivity (67.1%), indicating it was the most effective at identifying those who may die. The Ryan score had the highest specificity (81.6%), indicating it was the best at identifying survivors.

Several factors could explain the lower AUROC values observed in this study. Firstly, our patient cohort had a larger TBSA burned than those in the original articles [3,4,5]. Higher TBSA is strongly associated with increased mortality risk, and this variation in burn severity may affect the performance of the scoring systems. Furthermore, our relatively small sample size may have limited statistical power.

Existing scores did not include other factors that have been shown to influence mortality in the literature. A Portuguese study has shown comorbidities and the Charlson comorbidity index to be an independent risk factor of mortality [11]. Other studies also have shown the relationship between outcome and comorbidities [12,13,14], and increased mortality in burn patients is observed in those with concomitant trauma injury [15,16] and those in need of mechanical ventilation [17].

Finally, a lot has changed in critical care management since the scores were developed. Advancements in burn care and resuscitation techniques may have contributed to the variation in results. Improvements in clinical management have reduced mortality, making it more challenging for traditional scoring systems to predict outcomes. For example, a United Kingdom study in burn patients from 1982 to 2008 showed significant improvement in survival during time in all age groups due to advancements in medicine, and better survival rates than those predicted if the Baux score was used [18]. The results indicate that, while the scores are good predictors of mortality, a prognosis should not depend solely on those.

When comparing the two time periods in our patient population, while mortality rates remained similar, a significant reduction in the duration of mechanical ventilation and LOS in the BICU was observed.

We would like to point out the main changes that have occurred. Prior to the protocol’s implementation, BICU specialists rotated on a daily or weekly basis. The lack of continuity in care may result in frequent treatment changes, and difficulties in monitoring patients’ clinical status. Also, each specialist had a different treatment plan, and sometimes treatment plans have changed daily with each specialist. The new structure, with a leading intensivist for an extended period (3–6 months), ensured continuity in care, better monitoring of changes in clinical status each day, and a consistent treatment plan. A leading intensivist had daily insight into the patient’s clinical progress and could recognize minor changes in clinical status, more confidently start discontinuation of mechanical ventilation, and oversee nurse care and physical therapy. LOS in the BICU (35.83 ± 35.53 days) and LOS in the hospital (55.36 ± 48.80 days) (is long for burn patients (, and that is why it could be important for the same person to monitor patients every day. The adoption of standardized treatment protocols may further contribute to improved outcomes. Although protocols are standard in modern intensive care, the adherence to protocols may be better if one leading anesthesiologist oversees patients and treatment plan, and that was the motivation behind this organizational change. The consistent approach to managing inhalation injuries, sedation with dexmedetomidine, and focus on earlier discontinuation of sedation may play a role in better outcomes. We are aware of our limitations, and the effect of one measure alone could not be determined, but raising questions about continuity of care in burn intensive care could be important and further evaluated.

Our study has several limitations that must be considered when interpreting the results. First, the retrospective design introduces potential errors in data collection. Another significant limitation is the relatively small sample size, which may reduce the generalizability of the findings. While we analyzed the impact of new protocols and organizational changes, the number of patients in two time periods differs, and real improvements will be evident in a couple of years with more patients treated.

## Figures and Tables

**Table 1:** Characteristics of patients, injury, and patient outcomes.

		All (n = 149)	Survived (n = 76)	Deceased (n = 73)
Age (years)	Mean ± SD	54.62 ± 19.38	49.87 ± 19.28	59.56 ± 18.33
	Median (IQR)	55.79 (18–92)	49.90 (18–91)	61.13 (19–92)
Sex	Male	114 (76.51%)	57 (75.00%)	57 (78.08%)
	Female	35 (23.49%)	19 (25.00%)	16 (21.92%)
TBSA burned (%)	Mean ± SD	42.98 ± 19.90	34.82 ± 14.05	51.47 ± 21.56
	Median (IQR)	35.00 (20–100)	30 (20–80)	50 (20–100)
Full thickness burns (n)		132 (88.59%)	64 (84.21%)	68 (93.15%)
Inhalational injury (n)		70 (46.98%)	28 (36.84%)	42 (57.53%)
Mechanical ventilation (n)		116 (77.85%)	46 (60.53%)	70 (95.89%)
Mechanical ventilation (days)	Mean ± SD	22.72 ± 23.38	20.33 ±17.78	24.30 ± 26.43
	Median (IQR)	14.00 (1–121)	14.00 (1–74)	14.00 (1–121)
LOS/TBSA	Mean ± SD		2.49 ± 1.61	
	Median (IQR)		2.00 (0.25–7.70)	
LOS BICU (days)	Mean ± SD	35.83 ± 35.53	40.80 ± 36.24	30.65 ± 34.26
	Median (IQR)	24.00 (0–197)	29.50 (2.00–197.00)	18.00 (0–148)
LOS hospital (days)	Mean ± SD	55.36 ± 48.80	78.83 ± 48.63	30.92 ± 35.28
	Median (IQR)	41 (0–246)	65.00 (19–246)	18.00 (0–168)

**Table 2:** Prognostic scores.

	Score (Mean ± SD)	AUROC	Sensitivity	Specificity	PPV	NPV
rBaux	105.69 ± 28.93	0.79	67.1	71.1	69.0	69.2
ABSI	9.26 ± 2.38	0.79	54.8	78.9	71.4	64.5
BOBI	4.32 ± 2.15	0.77	61.6	71.1	67.2	65.9
Ryan	1.30 ± 0.91	0.76	57.5	81.6	75.0	66.7

**Table 3:** Characteristics of patients, injury, and outcomes in two different time periods; first from 2016 to 2022, and second from 2023 to 2024.

		First Period (n = 114)	Second Period (n = 35)	
Age (years)	Mean ± SD	54.70 ± 20.16	54.34 ± 16.82	*p* = 0.7324
	Median (IQR)	59.47 (18–91)	52.97 (20–92)	
Sex	Male	84 (73.68%)	30 (85.71%)	*p* = 0.1420
	Female	30 (26.32%)	5 (14.29%)	
TBSA burned (%)	Mean ± SD	41.99 ± 18.63	46.19 ± 23.59	*p* = 0.6085
	Median (IQR)	35.00 (20–100)	40.00 (20–93)	
Full thickness burns (n)		100 (87.72%)	32 (91.43%)	*p* = 0.5460
Inhalational injury (n)		50 (43.86%)	20 (57.14%)	*p* = 0.1684
LOS/TBSA	Mean ± SD	2.49 ± 1.55	2.51 ± 1.85	
	Median (IQR)	2.03 (0.57–6.95)	1.89 (0.25–7.70)	
Mechanical ventilation (days)	Mean ± SD	25.22 ± 24.95	13.64 ± 13.29	*p* = 0.0149
	Median (IQR)	16.00 (1–121)	10 (1–53)	
LOS BICU (days)	Mean ± SD	40.56 ± 38.23	20.40 ± 17.82	*p* = 0.0022
	Median (IQR)	29.00 (0–197)	16.00 (0–67)	
LOS hospital (days)	Mean ± SD	58.61 ± 49.25	44.77 ± 46.41	*p* = 0.0700
	Median (IQR)	51.00 (0–246)	33 (0–177)	
Mortality	Survived	58 (50.88%)	18 (51.43%)	*p* = 0.9545
	Died	56 (49.12%)	17 (48.57%)	

## Data Availability

Data are available on request due to ethical and legal reasons.
